# Effect of the Mediterranean diet supplemented with nicotinamide riboside and pterostilbene and/or coconut oil on anthropometric variables in amyotrophic lateral sclerosis. A pilot study

**DOI:** 10.3389/fnut.2023.1232184

**Published:** 2023-09-22

**Authors:** Sandra Carrera-Juliá, José M. Estrela, Mario Zacarés, Mari Ángeles Navarro, María Jesús Vega-Bello, José Enrique de la Rubia Ortí, Mari Luz Moreno, Eraci Drehmer

**Affiliations:** ^1^Department of Nutrition and Dietetics, Catholic University of Valencia San Vicente Mártir, Valencia, Spain; ^2^Department of Physiology, University of Valencia, Valencia, Spain; ^3^Department of Basic and Transversal Sciences, Catholic University of Valencia San Vicente Mártir, Valencia, Spain; ^4^Department of Human Anatomy and Physiology, Catholic University of Valencia San Vicente Mártir, Valencia, Spain; ^5^Department of Nursery, Catholic University of Valencia San Vicente Mártir, Valencia, Spain; ^6^Department of Health and Functional Assessment, Catholic University of Valencia San Vicente Mártir, Valencia, Spain

**Keywords:** amyotrophic lateral sclerosis, nicotinamide riboside, pterostilbene, coconut oil, nutrition, anthropometry

## Abstract

Amyotrophic Lateral Sclerosis (ALS) is a chronic and progressive neurodegenerative disease that causes the death of motor neurons and alters patients’ body composition. Supplementation with the antioxidants nicotinamide riboside (NR) and pterostilbene (PTER) can combat associated oxidative stress. Additionally, coconut oil is an alternative energy substrate that can address mitochondrial dysfunction. The aim of the present study is to assess the impact of a Mediterranean Diet supplemented with NR and PTER and/or with coconut oil on the anthropometric variables of patients with ALS. A prospective, mixed, randomized, analytical and experimental pilot study in humans was performed through a clinical trial (registered with ClinicalTrials.gov under number NCT03489200) with pre- and post-intervention assessments. The sample was made up of 40 subjects categorized into four study groups (Control, Antioxidants, Coconut oil, and Antioxidants + Coconut oil). Pre- and post-intervention anthropometric assessments were carried out to determine the following data: weight, percentage of fat and muscle mass, skinfolds, body perimeters, Body Mass Index (BMI), Waste-to-Hip Index (WHI) and Waist-Height Ratio (WHR). Compared to the Control group, GAx significantly increased muscle mass percentage and decreased fat mass percentage, triceps, iliac crest, and abdominal skinfolds. GCoco significantly increased muscle mass percentage and decreased fat mass percentage, subscapular skinfolds, and abdominal skinfolds. GAx + coco significantly increased muscle mass percentage and decreased abdominal skinfolds. Therefore, our results suggest that the Mediterranean Diet supplemented with NR and PTER and the Mediterranean Diet supplemented with coconut oil (ketogenic diet) are the two nutritional interventions that have reported the greatest benefits, at anthropometric level.

## 1. Introduction

Amyotrophic Lateral Sclerosis (ALS) is a chronic and progressive neurodegenerative disease of the Central Nervous System (CNS), of a neuromuscular type, characterized by the degeneration and selective dysfunction of upper and lower motor neurons (1). It is associated with a degeneration of the neuromuscular junctions that lead to skeletal muscle atrophy (2), difficulty performing voluntary movements, decreased motor autonomy and impaired oral communication, swallowing and breathing (3). It occurs in adults aged between 55 and 65, with peak incidence between 50 and 75 (4), although cases have been identified in patients younger than 25 (5). There is a crude prevalence and incidence of ALS worldwide of 4.42 (95% CI 3.92–4.96) per 100,000 population and 1.59 (95% CI 1.39–1.81) per 100,000 person-years, respectively (6). Males are considered more at risk, with an incidence rate of 1.6 in males compared to 1.2 in females (7).

Scientific evidence has identified Oxidative Stress (OS) as one of the multiple pathogenic mechanisms contributing to the development and progression of the disease (8). In addition, a deficient antioxidant capacity of the organism that increases the disruption in redox homeostasis and motor neuron death has been described (9). OS is accompanied by mitochondrial dysfunction, impaired functioning of the enzyme superoxide dismutase 1 (SOD1), excitotoxicity caused by increased neurotransmitter glutamate, neuroinflammation, decreased nicotinamide adenine dinucleotide (NAD^+^) levels and difficulty replenishing them (10).

The precursor nicotinamide riboside (NR) has been scientifically proven to replenish NAD^+^ levels (11). NR (vitamin B3 or niacin) is a nucleoside made up of nicotinamide and a ribose group that can be found in vegetables, eggs, fish, milk and fortified products (12). Animal and human studies have shown that NR supplementation may be an effective and safe way to replenish NAD^+^ levels (13). There is also interest in pterostilbene (PTER) as an antioxidant treatment. PTER (trans-3,5-dimethoxy-4 hydroxystilbene) is a dimethylated natural stilbene comprising 1 hydroxyl group and 2 methoxy groups (14). It belongs to the family of polyphenols, and is present in fruits, vegetables, legumes, whole grains, seeds, nuts and extra virgin olive oil (15). It can activate metabolic pathways related to protection against OS, neuroinflammation, regulation of excitotoxicity and preservation of cognitive functions. For this reason, it could be a promising therapeutic strategy in diseases associated with OS and neurological damage, such as ALS (16).

Mitochondrial dysfunction involves a reduction in metabolic energy production (17) that compromises the supply of glucose and ATP to motor neurons and increases the risk of neurodegeneration (18), production of reactive oxygen species (ROS), oxidative damage and cell apoptosis (19). Consequently, it has been suggested that the activation of alternative metabolic pathways such as fatty acid beta oxidation could be a useful strategy to address the high energy demand of neuronal tissues (20). Furthermore, the synthesis of ketone bodies is an alternative energy source to the impaired glycolytic pathway (21). In addition, they act on metabolic pathways that regulate neuroinflammatory processes, glutamate regulation and excitotoxicity (22). Ketone bodies have shown anabolic and anti-catabolic effects in skeletal muscle, being especially relevant in ALS, due to the negative impact that this disease produces on muscle mass (23).

Medium chain triglycerides (MCT) are considered effective in the synthesis of ketone bodies since they require less energy and can be absorbed from the intestine to move through the portal vein directly to the liver without the need to circulate through the lymphatic system (24). Coconut oil has a nutritional composition characterized by a high contribution of MCT, representing up to 60–70% of total fat, the majority being caproic, caprylic, capric and lauric acids (25). Because of the above, nutritional supplementation with coconut oil could be a good way to promote the synthesis of ketone bodies as an alternative energy substrate to address the ineffectiveness of the glycolytic pathway identified in ALS (26).

Regrettably, there is no definitive cure for ALS and approved pharmacological therapies include Riluzol, Edaravone (Radicava^®^) (27), Relyvrio^®^ (Sodium phenylbutyrate-taurursodiol) (28) and Qalsody^®^ (Tofersen) (29). In addition to the negative impact that the multisystemic degeneration has on patient health, only 25% of patients affected by this disease live longer than 5 years after diagnosis and 5–10% longer than 10 (30). However, a healthy nutritional diet has been identified as increasing patients’ long-term survival and as being a determining factor in the evolution of the disease (31). Specifically, the Mediterranean Diet provides antioxidant and anti-inflammatory substances that help preserve nutritional status. In addition, this diet is a neuroprotective factor that prevents neuronal degeneration and decreases the risk of developing neurodegenerative diseases (32).

Based on the above, the aim of this study was to evaluate the effect of the Mediterranean Diet supplemented with the antioxidants nicotinamide riboside and pterostilbene and/or coconut oil on anthropometric variables in patients affected by ALS.

## 2. Materials and methods

A prospective, mixed, randomized, analytical and experimental pilot study in humans was performed through a clinical trial with pre- and post-intervention assessment.

### 2.1. Subjects

#### 2.1.1. Sample size

Prior to sample selection, the sample size was determined by considering the possible difficulties that might be encountered in establishing a large sample: e.g. displacement of patients residing in different Autonomous Communities of Spain and insufficient degree of motivation to adhere to the study, among others.

The pwr package was used included in the R programming environment. Calculations were carried out considering a one factor analysis of variance (ANOVA) and balanced groups, with a large effect size (Cohen’s f equal to 0.6), a statistical power equal to 0.8, and a significance level of 0.05. The variables of interest were fat mass and muscle mass percentages. It was estimated that nine participants per group would provide an appropriate size to determine differences in anthropometric variables between pre- and post-intervention measurements. Thus, it was estimated a minimum sample size of 36 subjects.

#### 2.1.2. Inclusion and exclusion criteria

In order to obtain the study sample, the Spanish Foundation for the “Fundación Española para el Fomento de la Investigación de la Esclerosis Lateral Amiotrófica” (FUNDELA) was contacted, with the aim of identifying potential participants. Sixty potential candidates residing in different Autonomous Communities of Spain were recruited.

**Figure 1:**
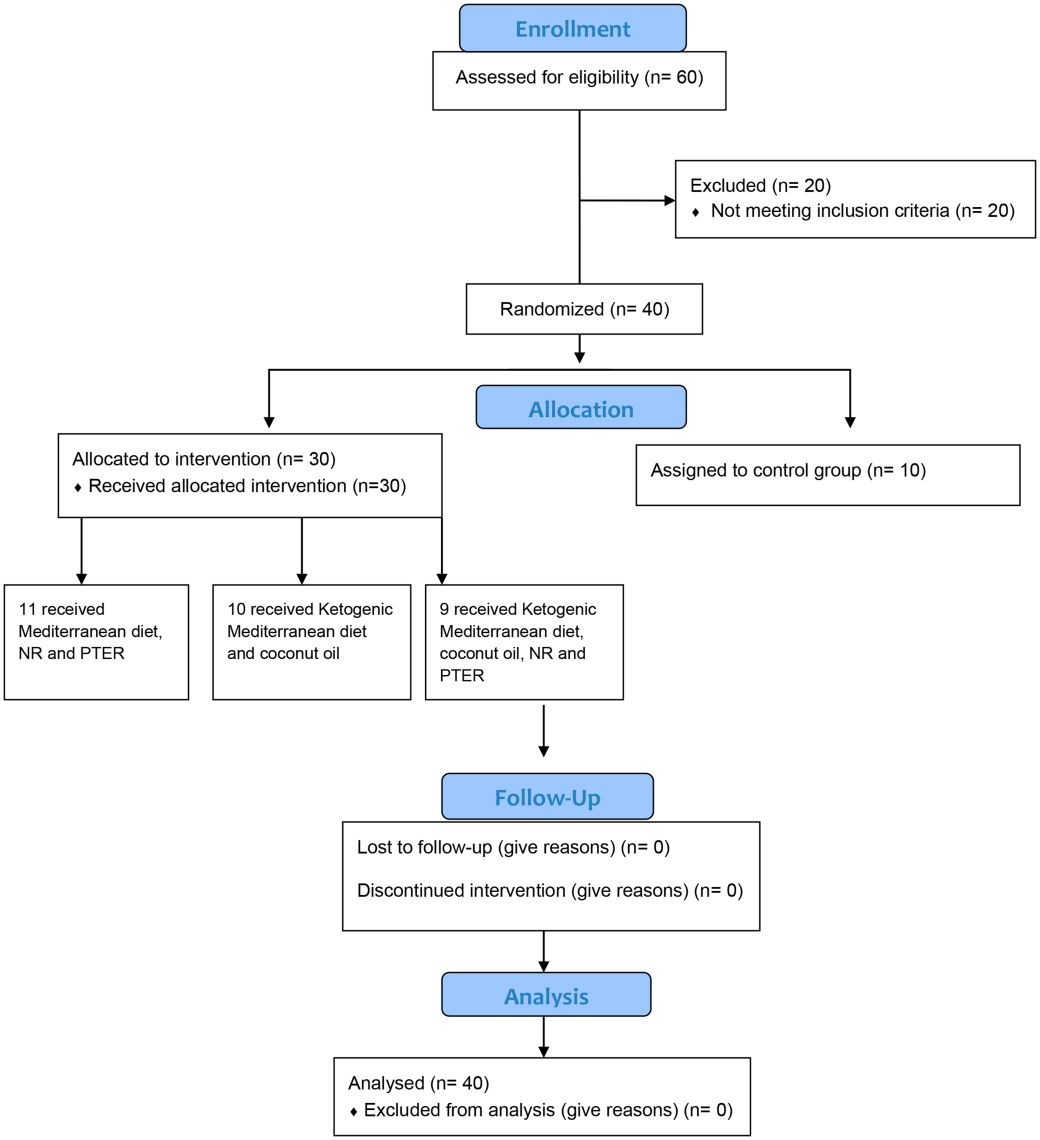
CONSORT Flow Diagram for the allocation of the sample.

The following inclusion criteria were applied: patients older than 18, diagnosed with ALS (spinal, bulbar or familial) with a minimum of six months’ evolution of the disease determined by the “Criterios de El Escorial” and able to eat their food orally. Exclusion criteria included: pregnant or lactating women; patients with tracheostomy, who need invasive or non-invasive ventilation; patients with evidence of alcohol and other drug use; undergoing gastrectomy; fully or partially consuming their food through Percutaneous Endoscopic Gastrostomy (PEG); had suffered a heart attack or present cardiac complications; infected with hepatitis B or C and/or Human Immunodeficiency Virus (HIV); present renal damage or creatine levels 2 times higher than normal and patients who present evidence of dementia. A sample of 40 male and female patients was obtained, as shown in the Consort Diagram (Figure 1) (33).

#### 2.1.3. Ethical concerns

This study was approved by the University of Valencia Institutional Review Board on Human Studies and all the procedures related to the participants were approved by the University of Valencia Ethics Committee under reference number H1479983999044. This study was registered with ClinicalTrials.gov under number NCT03489200. All the interventions carried out followed the guidelines established by the Declaration of Helsinki (34). Participants were provided with a written informed consent form after being informed of the procedures and the nature of the study.

#### 2.1.4. Intervention groups

A simple randomization methodology was followed. The 40 participants were distributed to configure the control group (GControl) and three experimental groups: antioxidant group (GAx), coconut oil group (GCoco) and antioxidant + coconut oil group (GAx + coco). Although patients were randomly allocated in the different groups based on the age parameter, we found no statistical difference among those groups. Moreover, the rest of parameters displayed in Table 1 (particularly, ALS duration, ALSFRS-R and body mass index) show that the patient population in the different groups reflects an acceptable homogeneity.

All the study groups underwent a pre-intervention clinical and dietary-nutritional anamnesis assessment. They also underwent pre- and post-intervention anthropometric assessments. Each group followed a meal plan along with its corresponding nutritional supplementation, with the exception of GControl, which did not receive any of the previous prescriptions and continued with their usual eating habits.

GAx: followed a Mediterranean diet + NR and PTER antioxidants.

GCoco: followed a Ketogenic Mediterranean diet + coconut oil.

GAx + coco: followed a Ketogenic Mediterranean diet + coconut oil + NR and PTER antioxidants.

All participants (n = 40) completed pre- (time = 0) and post-intervention (after 4 months) measurements and assessments.

### 2.2. Procedures

Procedures were carried out in clinical facilities located in the city of Valencia (Spain) and adapted to the study. Family members and/or caregivers were also involved, especially in those situations in which the patient had mobility or communication difficulties.

#### 2.2.1. Clinical and dietary-nutritional anamnesis

It was carried out a pre-intervention clinical anamnesis assessment on all study groups to characterize their individual clinical context. This procedure allowed us to collect certain sociodemographic and clinical variables: place of residence, age, ALS clinical phenotype, onset of symptoms, date of diagnosis, personal history, family history, among others.

It was carried out a dietary-nutritional anamnesis using a Food Frequency Questionnaire (FFQ) (35) and a food diary. A characterization of the usual eating habits of each patient was obtained. This gave provided an understanding of how frequently per week or month food was consumed, the number of meals per day, ingredients included in the meals and how dishes were prepared. This information was considered during the preparation of the meal plan and to individualize the diet to the particularities of each patient in terms of frequency of consumed foods, food preferences and/or aversions, among others.

#### 2.2.2. Anthropometric assessment

It was carried out pre- and post-intervention anthropometric assessments in all study groups, before and four months after the intervention. In both measurements, the anthropometric method followed was the protocol established by the International Society for the Advancement of Kinanthropometry (ISAK) and accepted by the Spanish Group of Kinanthropometry (GREC) (36). Measurements were taken by an ISAK level III certified anthropometrist.

Body weight was measured with a portable clinical scale, SECA model with a capacity of 150–200 kg and precision of 100 g. In cases of reduced mobility, an electronic chair-type scale was used, model SECA 954 with a maximum capacity of 300 kg and precision of 100 g. In order to measure height, it was used a SECA 220 Hamburg, Germany stadiometer, with a precision of 0.1 cm, after locating the Frankfort plane. A mechanical calliper, Holtain LTD Crymych UK model, with a precision of 0.2 mm and a measurement range of 0 to 48 mm, was used in order to measure skinfolds (triceps, subscapular, iliac and abdominal crest). Body perimeters (waist and hip) were taken using a flexible steel anthropometric tape, model Lufkin W606ME. A bicondylar pachymeter, Holtain model, was used for small bone diameters (humerus, bistyloid, and femur), with a minimum precision of 1 mm, and a measurement range from 0 to 140 mm, for the measurement of body diameters.

The anthropometric indexes of Body Mass Index (BMI), Waist-Hip Index (WHI) and Waist-Height Ratio (WHR) were determined. Body compartments were calculated as the percentage of fat mass using the Faulkner equation (37). Bone weight was calculated with the Rocha formula (38) and the Matiegka formula (39) was used to calculate muscle weight, which was used to obtain muscle mass percentage.

#### 2.2.3. Dietary plan

For the design of the dietary plans to be followed by the experimental groups (GAx, GCoco and Gax + coco), it was used the dietary-nutritional software “Nutrición y Salud” version 2.0 (University of Granada, Spain) and a weight guide for home measurements and usual consumption rations (40).

A seven-day dietary plan was developed, and the average weekly nutritional value was collected. The percentage distribution of macronutrients (carbohydrates, proteins and lipids) was adjusted to the nutritional objectives for the Spanish population established by “Sociedad Española de Nutrición Comunitaria” (SENC) (41). The contribution of micronutrients (vitamins and minerals) was adjusted taking into account the Dietary Reference Intakes (DRI) established by the “Federación Española de Sociedades de Nutrición, Alimentación y Dietética” (42) in 2010. The dietary plans were designed using the basic characteristics of the Mediterranean Diet. On certain occasions, the dietary plan had to be adapted to personal circumstances to facilitate adherence (texture and consistency adaptation in cases of dysphagia, food avoidance in cases of allergies or food intolerances). However, the caloric intake, the percentage distribution of macronutrients and the DRI of micronutrients were respected when this occurred.

##### 2.2.3.1. Dietary plan based on the Mediterranean diet

The Mediterranean Diet provided approximately 2,300 kcal distributed in five meals per day. It included 35 mL of extra virgin olive oil, distributed as follows: 5 mL at breakfast or morning snack, 15 mL at lunch and 15 mL at dinner. Table 2 shows the average nutritional contribution of this meal plan.

**Table 2:** Mean nutritional contribution of the dietary plan based on a Mediterranean diet.

Nutrient	Average weekly contribution	DRI
Energy (kcal)	2,339	1875–3,000
Carbohydrates (%)	51	50–55
Proteins (%)	16	10–20
Lipids (%)	33	30–35
Monounsaturated fatty acids (%)	16.58	20
Polyunsaturated fatty acids (%)	7.16	5
Saturated fatty acids (%)	5.64	7–8
Cholesterol (mg)	209.19	300
Fiber (*g*)	43.63	25–35

The percentage distribution of macronutrients (carbohydrates, proteins and lipids) is expressed compared to the total caloric volume (TCV) of the dietary plan. The contribution of macronutrients, fiber, and cholesterol was adjusted to the nutritional objectives for the Spanish population stipulated in the “Sociedad Española de Nutrición Comunitaria” (SENC) consensus. DRI: Dietary Reference Intakes.

##### 2.2.3.2. Mediterranean dietary plan supplemented with coconut oil

Comprised a ketogenic Mediterranean dietary plan that provided 2,300 kcal divided into five meals per day. It included a total of 60 mL of coconut oil distributed as follows: 20 mL at breakfast, 20 mL at lunch and 20 mL at dinner, or 30 mL at lunch and 30 mL at dinner. Table 3 shows the mean nutritional contribution.

**Table 3:** Mean nutritional contribution of the dietary plan based on a ketogenic Mediterranean diet.

Nutrient	Average weekly contribution	DRI
Energy (kcal)	2,342	1875–3,000
Carbohydrates (%)	40	50–55
Proteins (%)	20	10–20
Lipids (%)	40	30–35
Monounsaturated fatty acids (%)	7.10	20
Polyunsaturated fatty acids (%)	5.26	5
Saturated fatty acids (%)	23.21	7–8
Cholesterol (mg)	266.03	300
Fibre (g)	38.59	25–35

The percentage distribution of macronutrients (carbohydrates, proteins and lipids) is expressed compared to the total caloric volume (TCV) of the dietary plan. The contribution of macronutrients, fibre and cholesterol was adjusted to the nutritional objectives for the Spanish population stipulated in the “Sociedad Española de Nutrición Comunitaria” (SENC) consensus. DRI: Dietary Reference Intakes.

Regarding the coconut oil, it was recommended heating it in a bain-marie to facilitate ingestion. The option of eating it directly or mixing it with juice was given to improve palatability.

The contribution of saturated fatty acids from coconut oil predominates in this dietary plan, as it provides up to 85.5 grams per 100 mL. On the other side, the contribution of monounsaturated fatty acids and polyunsaturated fatty acids is lower compared to other types of oils, such as olive oil. For this reason, the lipid profile of this meal plan did not comply with the DRI, since these have not been established based on a meal plan that provides coconut oil as the main fat.

##### 2.2.3.3. Nutritional supplementation with NR and PTER antioxidants

A combination of 1-(beta-D-ribofuranosyl) nicotinamide chloride and 3,5-dimethoxy-4′-hydroxy-trans-stilbene was used from compound EH301. It was administered in a capsule at a dose of 15 mg NR and 2.5 mg PTER/kg body weight/day. One capsule of compound EH301 was provided for every 10 kg of participant body weight. The total number of EH301 capsules was distributed into two doses: half in the morning (mid-morning) and the other half in the afternoon. There were taken with water and by the GAx and GAx + coco groups.

#### 2.2.4. ALSFRS-R test

The revised ALS functional rating scale (ALSFRS-R test) was performed in all study groups at baseline evaluation and 4 months after the intervention. It is a sensitive, accurate and reproducible scale, which assesses functional ability taking into account the domains of impairment: bulbar, upper limb, lower limb and respiratory (43).

#### 2.2.5. Monitoring

The data collected in the pre-intervention clinical, dietary-nutritional anamnesis and anthropometric assessments were analyzed to prepare an individualized report on nutritional status. Each patient was provided with the corresponding dietary plan and nutritional supplementation, along with personalized dietary recommendations to improve eating habits.

After starting the intervention, individualized follow-up phone-call were established to discuss any issues and talk about possible difficulties in swallowing, taste changes or tolerance to textures. These monitoring sessions were used to make necessary adaptations in the dietary plan to ensure adherence.

### 2.3. Statistical analysis

The statistical analysis was carried out using R software. It was performed a descriptive analysis of all the dependent variables of the study according to the group and when the assessments were carried out. The results are presented as mean ± standard deviation or as the number of patients compared to the total sample number. To determine the effect of the diets on the anthropometric variables analyzed, an ANOVA test was applied to the pre-post differences. The normality of the variables was analyzed using the Shapiro–Wilk test and Q-Q plots (quantile comparison). Homoscedasticity was checked using Levene’s test. When the studied variable did not meet the normality and homoscedasticity criteria, the Kruskal-Wallis non-parametric test was applied. All effects were considered significant when a value of *p* ≤0.05 was obtained. When the existence of differences between the study groups was determined, a Tukey Post-Hoc Analysis was applied in order to identify the groups with statistically significant differences.

## 3. Results

### 3.1. Demographic and clinical characteristics of the different study groups

Table 1 describes the demographic and clinical characteristics the beginning of the study.

**Table 1:** Demographic and clinical characteristics of the study sample at the beginning of the study.

Variable	GControl (*n* = 10)	GAx (*n* = 11)	GCoco (*n* = 10)	GAx + coco (*n* = 9)
*Sex, n*				
Males	5	8	5	7
Females	5	3	5	2
Age (years), Mean ± SD	48.70 ± 6.50	56.73 ± 12.13	57.90 ± 9.84	55.33 ± 9.97
Age (years), min-max	37–58	38–80	44–78	45–70
*Origin, n*				
Valencian community	6	2	6	2
Other communities	4	9	4	7
*Clinical phenotype, n*				
Spinal ALS	6	7	6	4
Bulbar ALS	3	4	1	5
Familial ALS	1	0	3	0
Diagnosis time (years), min-max	0–4	0–3	0–4	1–2
ALS duration (years), min-max	2–6	1–6	1–5	2–8
ALSFRS-R	40.30 ± 5.10	39.10 ± 4.90	40.40 ± 5.20	40.70 ± 5.40
Weight (kg), mean ± SD	63.33 ± 6.06	68.56 ± 6.61	70.49 ± 12.71	69.69 ± 8.08
Height (cm), mean ± SD	164.3 ± 9.12	170.55 ± 7.09	167.30 ± 10.11	169.00 ± 8.83
BMI (kg/m^2^), mean ± SD	23.61 ± 2.23	23.60 ± 1.85	25.01 ± 2.66	24.44 ± 3.52
Fat mass (%), mean ± SD	21.74 ± 6.37	20.60 ± 8.63	26.00 ± 6.57	19.03 ± 3.89
Muscle mass (%), mean ± SD	34.75 ± 6.04	33.94 ± 6.65	31.68 ± 3.87	35.77 ± 3.68

GControl, Control group; GAx, Antioxidant group; GCoco, Coconut oil group; GAx + coco, Antioxidant group and coconut oil; ALS, Amyotrophic Lateral Sclerosis; ALSFRS-R, revised ALS functional rating scale; SD, Standard Deviation; BMI, Body Mass Index; max, maximum; min, minimum; *n*: sample number. The descriptive analysis values are shown as sample number, mean ± standard deviation, and minimum-maximum interval. The time to diagnosis (years) refers to the time elapsed from the onset of symptoms until diagnosis, with the minimum time being 0 years (diagnosis confirmed the same year as onset of symptoms) and the maximum time being four years (diagnosis four years from onset of symptoms). The duration of ALS (years) refers to the duration of the disease since identification of the onset of symptoms by the patient, until the time of the study, presenting the minimum and maximum time elapsed. The data available in the clinical file of each patient were used to determine the mean time elapsed between the onset of symptoms and the moment the disease was diagnosed, in addition to the information provided during the interview and clinical anamnesis. This allowed to determine whether the diagnosis was made in the same year as the onset of symptoms or whether it was sometime later.

The sample comprised 40 people, 25 males (62.5%) and 15 females (37.5%). The mean age was 54.7 years, with an age range of 37–80. The youngest mean age was observed in GControl (48.70 ± 6.50), while the oldest mean age was seen in GCoco (57.90 ± 9.84). GAx presented the widest age range (38–80 years). 14% of the sample (16 subjects) resided in the Valencian Community, while the remaining 60% (24 subjects) came from other Autonomous Communities of Spain.

The predominant clinical ALS phenotype was spinal, identified in 23 subjects (57.5%). The time elapsed from the onset of symptoms to the time of this study ranged from a minimum of one year to a maximum of eight years. The GAx + coco presented the widest range of disease duration, between two and eight years.

At the beginning of the study the scores obtained in ALSFRS-R were: for GControl 40.30 ± 5.10; for GAx 39.10 ± 4.90; for GCoco was 40.40 ± 5.20; and for GAx + coco 40.70 ± 5.40. These values do not show significant differences, so the functional capacity of the four groups was similar at the beginning of the study. Likewise, the study groups were homogeneous according to weight (*p* = 0.166) and BMI (*p* = 0.551) at the beginning of the study. Regarding body weight, the total sample presented a mean weight (kg) of 67.99 ± 8.85 and a mean height (cm) of 167.83 ± 8.79. Mean BMI (kg/m^2^) was 24.14 ± 2.56. According to the classification scale (44), this was normal weight. The study groups were homogeneous regarding fat mass (*p* = 0.141) and muscle mass (*p* = 0.387) percentages. The total sample presented a mean fat mass (%) of 21.88 ± 6.94. Regarding the muscle mass percentage, the total sample presented an average equivalent to 33.99 ± 5.31.

### 3.2. Effect of nutritional intervention on anthropometric variables

#### 3.2.1. Analysis of weight, fat mass, and muscle mass variation

Table 4 shows the initial and final values of body weight, fat mass and muscle mass in the four study groups.

**Table 4:** Analysis of the variation in body weight, fat mass, and muscle mass as a function during the study in the different groups.

Variable	Group (*n*)	Pre-intervention assessment Mean ± SD	Post-intervention assessment Mean ± SD	Post – Pre-intervention assessments Mean ± SD	*p*-value
Weight (Kg)	GControl (10)	21.74 ± 6.37	23.30 ± 7.11	1.56 ± 1.14	0.5459
	GAx (11)	20.60 ± 8.63	18.19 ± 5.83	−2.41 ± 3.56	
	GCoco (10)	26.00 ± 6.57	24.16 ± 6.00	−1.84 ± 2.93	
	GAx + coco (9)	19.03 ± 3.89	18.37 ± 3.84	−0.66 ± 1.00	
Fat mass (%)	GControl (10)	21.74 ± 6.37	23.30 ± 7.11	1.56 ± 1.14	0.0049
	GAx (11)	20.60 ± 8.63	18.19 ± 5.83	−2.41 ± 3.56	
	GCoco (10)	26.00 ± 6.57	24.16 ± 6.00	−1.84 ± 2.93	
	GAx + coco (9)	19.03 ± 3.89	18.37 ± 3.84	−0.66 ± 1.00	
Muscle mass (%)	GControl (10)	34.75 ± 6.04	33.14 ± 6.26	−1.61 ± 1.12	0.0001
	GAx (11)	33.94 ± 6.65	35.35 ± 4.83	1.41 ± 2.00	
	GCoco (10)	31.68 ± 3.87	32.78 ± 3.53	1.10 ± 1.38	
	GAx + coco (9)	35.77 ± 3.68	36.53 ± 3.77	0.76 ± 1.06	

GControl, Control group; GAx, Antioxidant group; GCoco, Coconut oil group; GAx + coco, Antioxidant group and coconut oil; SD, Standard deviation; n, sample number. Descriptive analysis values are shown as Mean ± SD. All effects are considered significant with value of *p* ≤ 0.05.

**Figure 2:**
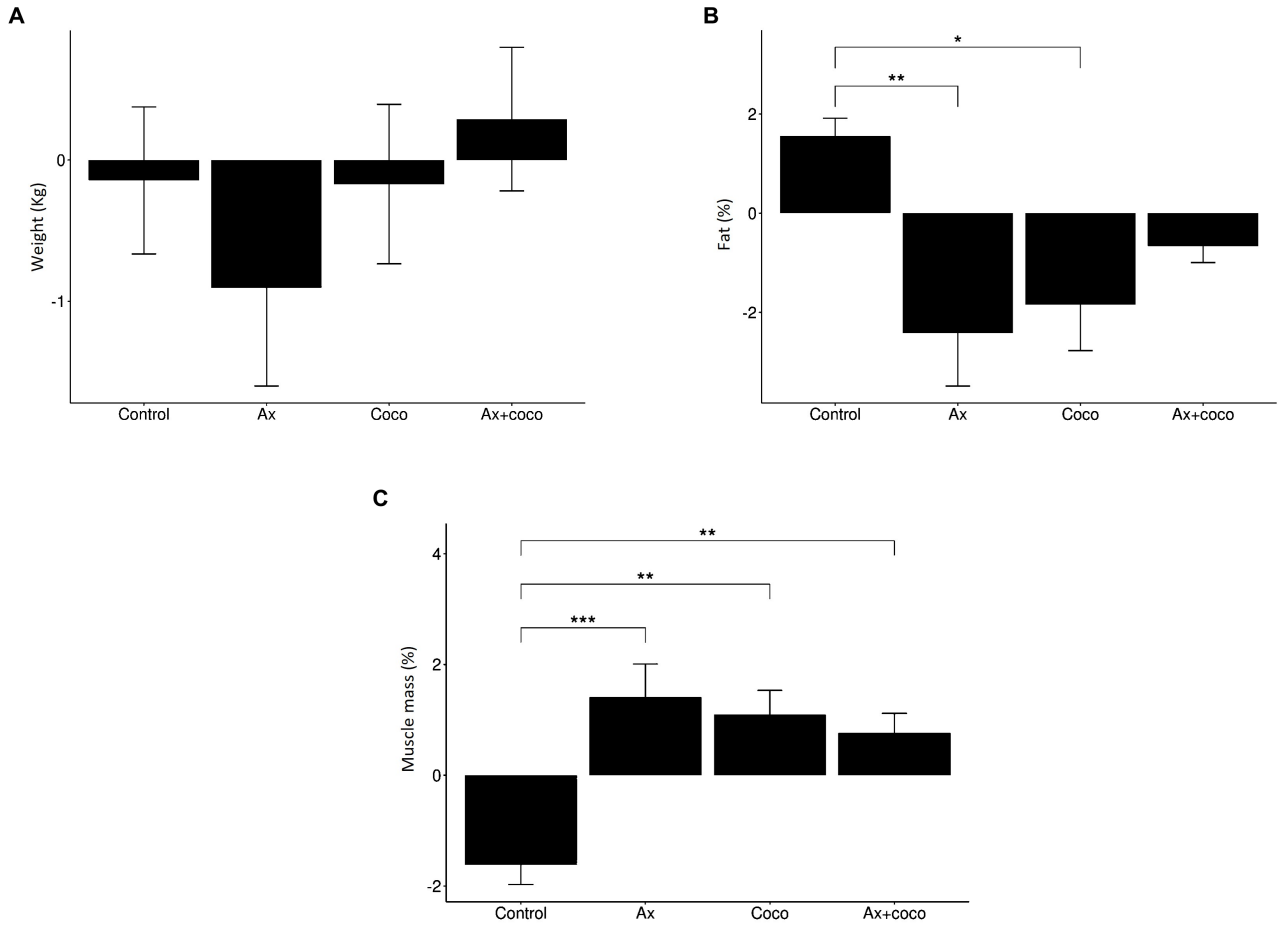
Variation in weight **(A)**, fat mass percentage **(B)** and muscle mass percentage **(C)** depending on study group. The statistical difference is indicated as: ***p ≤ 0.001 **p ≤ 0.01; *p ≤ 0.05.

The weight analysis (Figure 2A) shows that the study groups presented a similar behavior, obtaining very slight weight variations, with no significant changes. The GAx presented the greatest weight loss, while GAx + coco was the only group that showed a slight weight gain. No statistically significant differences were identified (*p* = 0.5459). Fat mass percentage (Figure 2B) decreased in all study groups, except for GControl, which presented an increase in this anthropometric variable. The greatest loss, (2.41%) was observed in GAx, followed by GCoco (1.84%) and GAx + coco (less than 1%). A statistically significant decrease in the fat mass percentage was identified in GAx and GCoco compared to GControl (*p* = 0.0045; *p* = 0.0216, respectively). There was a statistically significant increase in muscle mass percentage (Figure 2C) in GAx (*p* = 0.0002), GCoco (*p* = 0.0011) and GAx + coco (*p* = 0.0060), compared to GControl, which was the only group that showed a decrease. GAx had the highest muscle gain (1.41%), followed by GCoco (1.10%), and GAx + coco (0.76%).

#### 3.2.2. Anthropometric skinfold variation analysis

**Figure 3:**
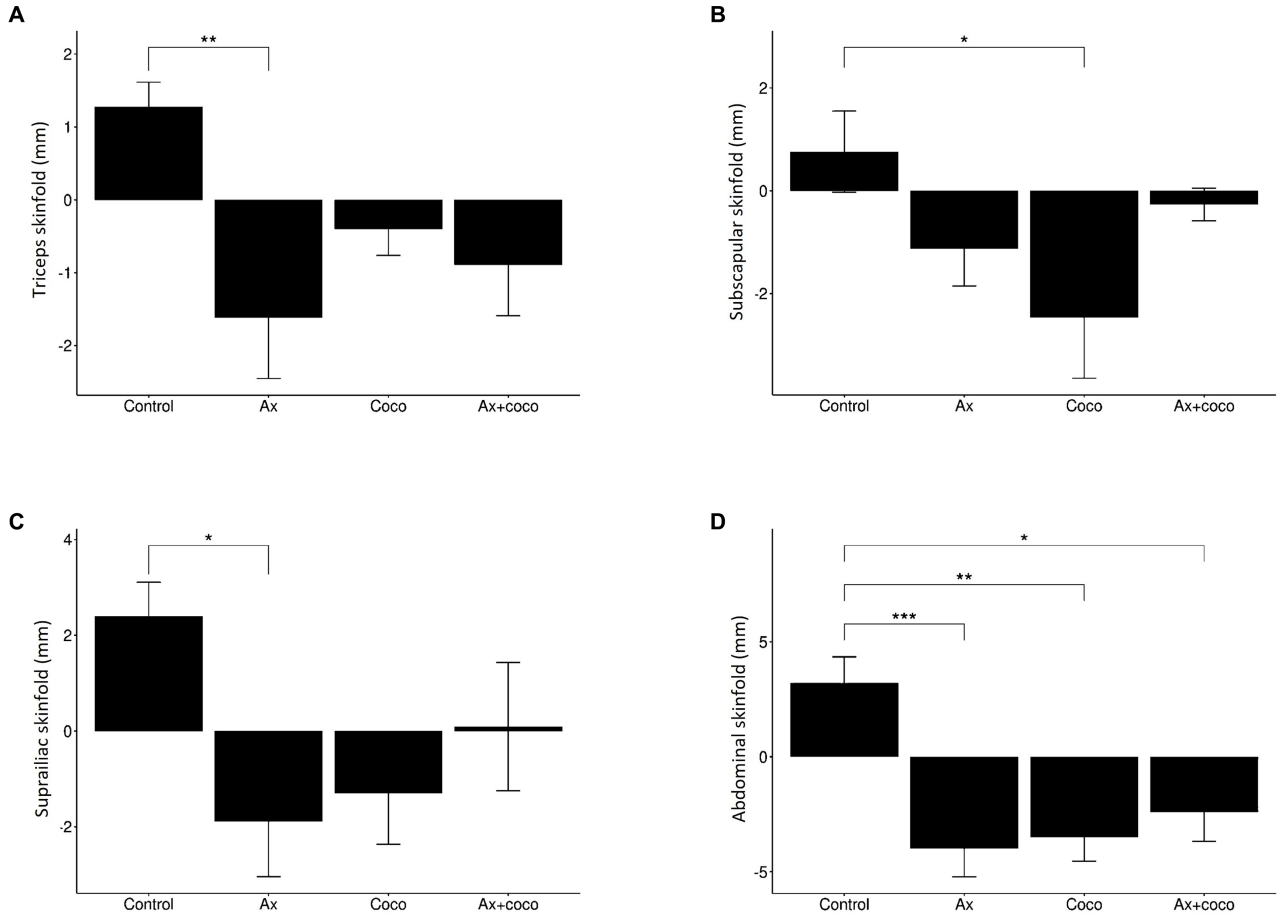
Variations of the triceps **(A)**, subscapular **(B)**, iliac crest **(C)** and abdominal **(D)** skinfolds according to study group. The statistical difference is indicated as: ***p ≤ 0.001 **p ≤ 0.01; *p ≤ 0.05.

All the study groups presented a decrease in triceps skinfold (Figure 3A), except for GControl, in which there was an increase. GAx showed the greatest decrease in this skinfold, being equivalent to 1.62 mm, which was statistically significant (*p* = 0.0077) when compared to GControl. A similar result was obtained in the subscapular skinfold measurements (Figure 3B): GAx, GCoco, and GAx + coco presented a decrease, while there was an increase in the GControl. A 2.46 mm decrease in the skinfold was seen in the GCoco that was statistically significant (*p* = 0.0439). Regarding the iliac crest skinfold (Figure 3C), GAx presented the greatest decrease of 1.89 mm, which was statistically significant (*p* = 0.0331) compared to GControl. The abdominal skinfold (Figure 3D) showed the highest number of significant effects. The greatest decrease was seen in GAx (4 mm), followed by GCoco (3.50 mm) and GAx + coco (2.40 mm). However, GControl presented an increase of 3.20 mm in this variable. The identified decrease was significant in GAx (*p* = 0.0004), GCoco (*p* = 0.0015) and GAx + coco (*p* = 0.0115) compared to GControl.

#### 3.2.3. Analysis of variation of body perimeters, anthropometric indexes, and functional capacity

The results related to the analysis of body perimeters and anthropometric indexes are shown in Table 5.

**Table 5:** Analysis of the variation of body perimeters, anthropometric indexes and ALSFRS-R depending on time and study group.

Variable	Group (*n*)	Pre-intervention assessment Mean ± SD	Post-intervention assessment Mean ± SD	Post – Pre-intervention assessments Mean ± SD	*p*-value
Waist (cm)	GControl (10)	84.50 ± 11.08	85.25 ± 11.36	0.75 ± 2.14	0.1266
	GAx (11)	88.82 ± 9.63	86.18 ± 9.94	−2.64 ± 3.44	
	GCoco (10)	92.95 ± 9.30	92.40 ± 10.85	−0.55 ± 3.89	
	GAx + coco (9)	89.61 ± 13.71	87.11 ± 10.55	−2.5 ± 4.70	
Hip (cm)	GControl (10)	94.92 ± 5.12	95.56 ± 5.61	0.64 ± 1.08	0.3427
	GAx (11)	96.00 ± 5.80	96.00 ± 4.45	0.00 ± 2.65	
	GCoco (10)	101.50 ± 5.08	100.55 ± 4.74	−0.95 ± 1.60	
	GAx + coco (9)	96.80 ± 3.70	96.22 ± 3.19	−0.58 ± 2.42	
BMI (kg/m^2^)	GControl (10)	23.61 ± 2.23	23.54 ± 2.37	−0.07 ± 0.59	0.5703
	GAx (11)	23.60 ± 1.85	23.29 ± 1.60	−0.31 ± 0.80	
	GCoco (10)	25.01 ± 2.66	24.95 ± 2.35	−0.06 ± 0.63	
	GAx + coco (9)	24.44 ± 3.52	24.54 ± 3.44	0.10 ± 0.56	
WHI	GControl (10)	0.89 ± 0.11	0.89 ± 0.11	0.00 ± 0.02	0.1255
	GAx (11)	0.93 ± 0.13	0.90 ± 0.13	−0.03 ± 0.03	
	GCoco (10)	0.92 ± 0.11	0.92 ± 0.13	0.00 ± 0.04	
	GAx + coco (9)	0.92 ± 0.12	0.90 ± 0.10	−0.02 ± 0.04	
WHR	GControl (10)	0.51 ± 0.07	0.52 ± 0.07	0.01 ± 0.01	0.1300
	GAx (11)	0.52 ± 0.07	0.51 ± 0.06	−0.01 ± 0.02	
	GCoco (10)	0.56 ± 0.04	0.55 ± 0.05	−0.01 ± 0.02	
	GAx + coco (9)	0.53 ± 0.09	0.52 ± 0.07	−0.01 ± 0.03	
ALSFRS-R	GControl (10)	40.30 ± 5.10	34.80 ± 3.00*	−5.50 ± 7.60	
	GAx (11)	39.10 ± 4.90	41.10 ± 2.90	2.00 ± 4.00	
	GCoco (10)	40.40 ± 5.20	39.90 ± 3.30	−0.50 ± 2.40	
	GAx + coco (9)	40.70 ± 5.40	39.50 ± 4.10	−1.20 ± 2.50	

GControl, Control group; GAx, Antioxidant group; GCoco, Coconut oil group; GAx + coco, Antioxidant group and coconut oil; BMI, body perimeters, Body Mass Index, WHI; Waste-to-Hip Index, WHR, Waist-Height Ratio; ALSFRS-R, revised ALS functional rating scale; SD, Standard deviation; n, sample number. Descriptive analysis values are shown as Mean ± SD. * *p* ≤ 0.05.

Regarding waist circumference (Table 5), a decrease was observed in GAx, GCoco and GAx + coco, with GAx presenting the greatest decrease. However, no statistically significant effects were identified (*p* = 0.1266). The variations of hip circumference (Table 5) were very slight, and no statistically significant effects were identified (*p* = 0.3427). When comparing the different study groups, the anthropometric indices (BMI, WHI and WHR) (Table 5) did not show an interaction effect. Regarding BMI, a similar behavior was observed in all groups, with very slight variations between the pre- and post-intervention assessments. A decrease in GAx and GAx + coco was seen in the WHI, while no variations were observed in GCoco and GControl. A decrease in the WHR was identified in GAx, GCoco and GAx + coco. On the contrary, GControl was the only group that obtained an increase in this variable.

In relation to the ALSFRS-R score, a statistically significant decrease (−5.50 ± 7.60) was observed in GControl. A slight decrease (−0.50 ± 2.40) was found in GCoco. A decrease equivalent to −1.20 ± 2.50 was observed in GAx + coco. In contrast, GAx was the intervention group with an increase equal to 2.00 ± 4.00.

## 4. Discussion

An adequate diet is important for ALS patients, since it ensures the nutritional needs and can minimizes the impact that the disease has on the nutritional status (45). It is suspected that the Mediterranean Diet may be beneficial since it includes foods that are rich in antioxidants, which have shown potential neuroprotective effect (46). Consequently, *t* is also thought that additional nutritional supplementations may exert further benefits The effects of both strategies require further exploration, which is why this present study conducted an analysis of the variation of anthropometric parameters.

We based our study on a sample of patients who had a stable weight, which is beneficial because a decrease in body weight is common in ALS patients and associate with progression, worse prognosis and increased mortality (47). Since the weight did not change, no significant changes were identified in associated variables such as BMI. This parameter remained constant in all groups (Table 5), being indicative of a normal weight. This is a positive finding because a BMI less than 18.5 kg/m^2^ or greater than 35 kg/m^2^ is associated with lower survival rates (48). Moreover, maintenance of BMI is indicative of good nutritional status and better prognosis of ALS, as it is associated with increased energy reserve, which is indispensable to compensate the increased caloric needs due to hypermetabolism (49). However, it is unclear that BMI can act as an anthropometric indicator due to its high specificity (97%) and low sensitivity (42%), such that its analysis may not specifically address the impact on body compartments (50). Because of this, fat and muscle mass were analyzed (Table 4; Figure 2).

In the analysis of the anthropometric skinfolds of the triceps, subscapular, iliac crest, and abdominal, a decrease that contributed to the reduction of body fat mass was observed (Figure 3). Furthermore, we found that GAx, GCoco and GAx + coco induced a decrease in the fat mass percentage compared to GControl (Figure 2), which could be considered a protective factor for ALS since a decrease in body fat is related to a decrease in proinflammatory cytokines, i.e., IL-6 or TNF-α, both involved in the pathogenic mechanism of inflammation that underlies the disease (51).

The decrease in fat mass percentage in GAx could be the consequence of following a Mediterranean Diet (52). It has been reported in animals models that polyphenols such as PTER also act on adipose tissue (53) by reducing lipogenesis and increasing fatty acid oxidation in the liver (54). Oral administration of MCT (coconut oil in our case) increases adipose tissue signaling (55) and lipolysis; two which may explain the decrease in fat mass (56). Coconut oil MCTs have a high oxidation rate and are used as an energy source instead of being stored in adipose tissue. Even so, further research is needed as these findings came from studies carried out in mice. Moreover, the discrepancy regarding the role of fat mass in ALS persists since some authors have indicated that adipose tissue could play a beneficial role in this disease and improve in survival rates (57).

ALS is associated with increased muscle catabolism, a factor that hinders the maintenance and synthesis of muscle (49). Despite this, all groups (GAx, GCoco, and GAx + coco) show an increase in their muscle mass percentage (Table 4; Figure 2), a positive result as a higher muscle mass percentage is directly associated with a slower rate of disease progression (58). Loss of NAD^+^ homeostasis promotes skeletal muscle degeneration (59), but NR supplementation restores its levels by activating the sirtuins, which is associated with an improvement of the oxidative capacity of the muscle, reducing the risk of loss of muscle mass (60). This could be why GAx presented the greatest increase in muscle mass, although we must note here that the effects of NR consumption on skeletal muscle in humans has not yet been completely proven (61). Moreover, adherence to the Mediterranean Diet followed by GAx is a protective factor for muscle mass, especially in middle-aged adults and during the ageing process (62, 63). Some studies have reported that the consumption of coconut oil favors the increase of lean mass (64). The underlying mechanism could be explained taking into account that saturated fatty acids contribute to the increase in ketone body levels that offset mitochondrial dysfunction and inefficient energy production, generating a positive impact on metabolism (65, 66) and facilitating energy bioavailability for muscle anabolic processes (67). The increase in muscle mass percentage the GCoco (Table 4; Figure 2) concurs with a previous study carried out by our research group on Multiple Sclerosis, where a sample of patients followed a Mediterranean Diet supplemented with coconut oil (68, 69). It should be added that adherence to a Mediterranean Diet is related to a good state of muscle mass, as this dietary pattern provides proteins necessary for the synthesis of muscle mass (70). On the contrary, GControl showed a decrease in muscle mass percentage (Table 4; Figure 2), a fact that may be due to the absence of a dietary plan and nutritional supplementation (71). Specifically, the GControl showed a worsening in body composition, manifested by an increase in fat mass and a decrease in muscle mass. These effects are typically associated with ALS progression. Indeed, the result observed in the body composition may serve as a prognostic factor and provide guidance for nutritional management in ALS patients (72).

Despite the absence of significant effects on body perimeters, it should be highlighted that the decrease in waist circumference is associated with less abdominal adiposity (Table 5). This decrease could be associated with the decrease observed in the abdominal skinfold (Figure 3), which presented the greatest number of significant effects, including a statistically significant decrease in GAx, GCoco, and GAx + coco, compared to GControl. Specifically, this anthropometric skinfold is measured in the abdomen, an area close to the waist. Consequently, the decrease observed in this skinfold would lead to a decrease in fat which could condition a decrease in volume manifested by a decrease in waist circumference. The accumulation of fat in this area has been reported to be associated with the production of proinflammatory cytokines leading to low-grade systemic inflammation (73), which has also been associated with neurodegeneration (74), decreased functional capacity (75) and cell apoptosis (76). Waist circumference is a marker of cardiovascular risk and insulin resistance (77), two factors that increase the risk of cardiovascular disease and diabetes, thus complicating patient prognosis.

All study groups presented a WHR greater than 0.5 (Table 5) in the pre-intervention assessment, a value considered unhealthy (78). The WHR continued to be greater than 0.5 in the post-intervention assessment (Table 5), but the decrease identified in the GAx, GCoco, and GAx + coco is related to a decrease in abdominal adiposity that results in a lower cardiovascular risk. It should be noted that the patients in this study had normal weight, low accumulation of adipose tissue in the abdominal region and slight variations in the hip circumference between the pre- and post-intervention assessments (Table 5). The above is probably why large changes in WHI were not observed.

In other clinical situations, anthropometric changes characterized by increased muscle mass and decreased fat mass are also accompanied by functional improvements. Specifically, it has been seen in women with breast cancer after a multidisciplinary rehabilitation program (79), in patients with cirrhosis after an exercise program (80) and in elderly women with sarcopenic obesity after a resistance training (81). However, in our study, with the exception of GControl which significantly worsened functional capacity after 4 months, the other 3 groups showed no change, with a worsening trend in the GAx + coco and GCoco groups and an improving trend in the GAx group (Table 5). These results suggest possible functional benefits of the interventions (greater in GAx) compared to the worsening observed in GControl.

However, it is important to note that the natural heterogeneity of the disease influences the interpretation of the evidences found in the present study and the limited sample size might be considered too small to confirm definitive conclusions. Besides, there is little available scientific evidence regarding the nutritional interventions used and their effect on anthropometric variables in humans affected by ALS, which makes consultation and direct comparison of results difficult. Moreover, each patient came from a different Spanish location and was attended by different health professionals depending on the correspondence hospital. Therefore, these professionals could have previously provided information on healthy nutritional habits that may influence the adherence to a dietary pattern. Specifically, the simultaneous prescription of a dietary plan together with nutritional supplementation does not allow to know which of the two strategies better contributes to the identified benefits or whether it is the sum of both. Added to this is the fact that ALS does not affect the limbs symmetrically, which is why different authors have suggested dual-energy X-ray absorptiometry (DEXA) or bone densitometry as a more precise methodology and one that could provide new findings in the study of ALS (82).

## 5. Conclusion

The Mediterranean Diet supplemented with NR and PTER and the Mediterranean Diet supplemented with coconut oil are the two nutritional interventions that seem to obtain greater anthropometric improvements in ALS patients. The limited sample size might preclude to reach definitive conclusions. Consequently, the preliminary data of this pilot study need to be implemented by a much larger clinical trial.

## Data availability statement

The original contributions presented in the study are included in the article/supplementary material, further inquiries can be directed to the corresponding author.

## Ethics statement

The studies involving humans were approved by the University of Valencia Institutional Review Board on Human Studies and all the procedures related to the participants were approved by the University of Valencia Ethics Committee under reference number H1479983999044. The studies were conducted in accordance with the local legislation and institutional requirements. The participants provided their written informed consent to participate in this study.

## Author contributions

JE, MM, and ED conceived and designed the study. SC-J, MN, and MV-B performed the experiments. SC-J and MZ analyzed the data. JO supervised the study. MM and SC-J wrote the paper. All authors contributed to the article and approved the submitted version.

## References

[1] Oskarsson B Gendron TF Staff NP. Amyotrophic lateral sclerosis: an update for 2018. Clin Proc. (2018) 93:1617–28. doi: 10.1016/j.mayocp.2018.04.007 30401437

[2] Valko K Ciesla L. Amyotrophic lateral sclerosis. Prog Med Chem. (2019) 58:63–7. doi: 10.1016/bs.pmch.2018.12.001 30879475

[3] Nijssen J Comley LH Hedlund E. Motor neuron vulnerability and resistance in amyotrophic lateral sclerosis. Acta Neuropathol. (2017) 133:863–5. doi: 10.1007/s00401-017-1708-8 28409282 PMC5427160

[4] Johansen M Svenstrup K Mortensen Ó Andorsdóttir GÁ Steig B Joensen P. Amyotrophic lateral sclerosis in the Faroe Islands—a genealogical study. Amyotr Late Scler Frontotemp Degener. (2020) 22:571–5. doi: 10.1080/21678421.2020.1813311 32885668

[5] Liu ZJ Lin HX Liu GL Tao QQ Ni W Xiao BG. The investigation of genetic and clinical features in Chinese patients with juvenile amyotrophic lateral sclerosis. Clin Genet. (2018) 92:267–3. doi: 10.1111/cge.13015 28429524

[6] Xu L Liu T Yao X Chen L Fan D Zhan L. Global variation in prevalence and incidence of amyotrophic lateral sclerosis: a systematic review and meta-analysis. J Neurol. (2020) 267:944–3. doi: 10.1007/s00415-019-09652-y 31797084

[7] Pradas J Puig T Rojas-García R Viguera ML Gich I Logroscino G. Amyotrophic lateral sclerosis in Catalonia: a population-based study. Amyotr Late Scler Frontotemp Degener. (2013) 14:278–3. doi: 10.3109/21678421.2012.749915 23286747

[8] Blasco H Garcon G Patin F Veyrat-Durebex C Boyer J Devos D. Panel of oxidative stress and inflammatory biomarkers in ALS: a pilot study. Can J Neurol Sci Le Journal Canadien Des Sciences Neurologiques. (2017) 44:90–5. doi: 10.1017/cjn.2016.284 27774920

[9] García ML Fernández A Solas MT. Mitochondria, motor neurons and aging. J Neurol Sci. (2013) 330:18–26. doi: 10.1016/j.jns.2013.03.019 23628465

[10] Yusuf M Khan M Robaian MA Khan RA. Biomechanistic insights into the roles of oxidative stress in generating complex neurological disorders. Biol Chem. (2018) 399:305–9. doi: 10.1515/hsz-2017-0250 29261511

[11] Cantó C Houtkooper RH Pirinen E Youn DY Oosterveer MH Cen Y. The NAD+ precursor nicotinamide riboside enhances oxidative metabolism and protects against high-fat diet induced obesity. Cell Metab. (2012) 15:838–7. doi: 10.1016/j.cmet.2012.04.022 22682224 PMC3616313

[12] Belenky P Racette FG Bogan KL McClure JM Smith JS Brenner C. Nicotinamide riboside promotes Sir2 silencing and extends lifespan via Nrk and Urh1/Pnp1/Meu1 pathways to NAD+. Cells. (2007) 129:473–4. doi: 10.1016/j.cell.2007.03.024 17482543

[13] Marinescu AG Chen J Holmes HE Guarente L Mendes O Morris M. Safety assessment of high-purity, synthetic nicotinamide riboside (NR-E) in a 90-day repeated dose Oral toxicity study, with a 28-day recovery arm. Int J Toxicol. (2020) 39:307–13. doi: 10.1177/1091581820927406 32715855

[14] Traversi G Fiore M Leone S Basso E Di Muzio E Polticelli F. Resveratrol and its methoxy-derivatives as modulators of DNA damage induced by ionising radiation. Mutagenesis. (2016) 31:433–1. doi: 10.1093/mutage/gew002 26819346

[15] Talhaoui N Gómez-Caravaca AM León L De la Rosa R Fernández-Gutiérrez A Segura-Carretero A. From olive fruits to olive oil: phenolic compound transfer in six different olive cultivars grown under the same agronomical conditions. Int J Mol Sci. (2016) 17:337. doi: 10.3390/ijms17030337 26959010 PMC4813199

[16] Carvalho JCT Fernandes CP Daleprane JB Alves MS Stien D Dhammika Nanayakkara NP. Role of natural antioxidants from functional foods in neurodegenerative and metabolic disorders. Oxidative Med Cell Longev. (2018) 2018:21459753. doi: 10.1155/2018/1459753 PMC6204203 30405873

[17] Martin LJ. Mitochondrial pathobiology in ALS. J Bioenerg Biomembr. (2011) 43:569–9. doi: 10.1007/s10863-011-9395-y 22083126 PMC4131252

[18] Barros LF San Martín A Sotelo-Hitschfeld T Lerchundi R Fernández-Moncada I Ruminot I. Small is fast: astrocytic glucose and lactate metabolism at cellular resolution. Front Cell Neurosci. (2013) 7 2014:27. doi: 10.3389/fncel.2013.00027 23526722 PMC3605549

[19] Rojas F González D Cortes N Ampuero E Hernández DE Fritz E. Reactive oxygen species trigger motoneuron death in non-cell-autonomous models of ALS through activation of c-Abl signaling. Front Cell Neurosci. (2015) 9:203. doi: 10.3389/fncel.2015.00203 26106294 PMC4460879

[20] Panov A Orynbayeva Z Vavilin V Lyakhovich V. Fatty acids in energy metabolism of the central nervous system. BioMed Res Int. (2014) 2014:472459. doi: 10.1155/2014/472459 24883315 PMC4026875

[21] Croteau E Castellano CA Richard MA Fortier M Nugent S Lepage M. Ketogenic medium chain triglycerides increase brain energy metabolism in Alzheimer’s disease. J Alzheimers Dis. (2018) 64:551–1. doi: 10.3233/JAD-180202 29914035

[22] Yudkoff M Daikhin Y Horyn O Nissim I Nissim I. Ketosis and brain handling of glutamate, glutamine, and GABA. Epilepsia. (2008) 49:73–5. doi: 10.1111/j.1528-1167.2008.01841.x PMC2722878 19049594

[23] Thompson JR Wu G. The effect of ketone bodies on nitrogen metabolism in skeletal muscle. Comp Biochem Physiol. (1991) 100:209–6. doi: 10.1016/0305-0491(91)90363-i 1799962

[24] St-Onge MP Ross R Parsons WD Jones PJH. Medium-chain triglycerides increase energy expenditure and decrease adiposity in overweight men. Obes Res. (2003) 11:395–2. doi: 10.1038/oby.2003.53 12634436

[25] Ghani NAA Channip A Chok Hwee Hwa P Ja'afar F Yasin HM Usman A. Physicochemical properties, antioxidant capacities, and metal contents of virgin coconut oil produced by wet and dry processes. Food Sci Nutr. (2018) 6:1298–06. doi: 10.1002/fsn3.671, PMID: 30065831 PMC6060898

[26] Norgren J Sindi S Sandebring-Matton A Kåreholt I Daniilidou M Akenine U. Ketosis after intake of coconut oil and Caprylic acid-with and without glucose: a cross-over study in healthy older adults. Front Nutr. (2020) 7:40. doi: 10.3389/fnut.2020.00040 32351966 PMC7175812

[27] Jaiswal MK. Riluzole and Edaravone: a tale of two amyotrophic lateral sclerosis drugs. Med Res Rev. (2019) 39:733–8. doi: 10.1002/med.21528 30101496

[28] Aschenbrenner DS. New drug approved for ALS. Am J Nurs. (2023) 123:22–3. doi: 10.1097/01.NAJ.0000911516.31267.67 36546382

[29] Blair HA. Tofersen: first approval. Drugs. (2023) 83:1039–43. doi: 10.1007/s40265-023-01904-6 37316681

[30] Longinetti E Fang F. Epidemiology of amyotrophic lateral sclerosis: an update of recent literature. Curr Opin Neurol. (2019) 32:771–6. doi: 10.1097/WCO.0000000000000730 31361627 PMC6735526

[31] Desport JC Preux PM Truong CT Courat L Vallat JM Couratier P. Nutritional assessment and survival in ALS patients. Amyotroph Lateral Scler Other Motor Neuron Disord. (2000) 1:91–6. doi: 10.1080/14660820050515386 11467055

[32] Petersson SD Philippou E. Mediterranean diet, cognitive function, and dementia: a systematic review of the evidence. Adv Nutr. (2016) 7:889–4. doi: 10.3945/an.116.012138 27633105 PMC5015034

[33] Schulz K Altman D Moher D. CONSORT 2010 statement: updated guidelines for reporting parallel group randomised trials. BMC Med. (2010) 8:18. 20334633 10.1186/1741-7015-8-18 PMC2860339

[34] World Medical Association. World medical association declaration of Helsinki: ethical principles for medical research involving human subjects. JAMA. (2013) 310:2191–4. doi: 10.1001/jama.2013.281053 24141714

[35] Trinidad-Rodríguez I Fernández Ballart J Cucó Pastor G Biarnés Jordà E ValV A. Validación de un cuestionario de frecuencia de consumo alimentario corto: reproducibilidad y validez. Nutr Hosp. (2008) 23:242–2. 18560701

[36] Stewart A Marfell-Jones M. International standards for anthropometric assessment. Lower Hutt: International Society for the Advancement of Kinanthropometry (2011). 65 p.

[37] Faulkner J. Physiology of swimming and diving In: Falls H, editor. Exercise physiology. Baltimore: Academic Press (1968)

[38] Rocha MSL. Peso ósseo do brasileiro de ambos os sexos de 17 a 25 años. Arquivos de Anatomía e Antropología. (1975) 1:445–1.

[39] Matiegka J. The testing of physical efficiency. Am J Phys Anthropol. (1921) 4:223–13. doi: 10.1002/ajpa.1330040302

[40] Carbajal A Sánchez-Muniz FJ. Guía de prácticas. Pesos de medidas caseras y raciones habituales de consumo In: García-Arias MT García-Fernández MC, editors. Nutrición y Dietética Secretariado de Publicaciones y Medios Audiovisuales. León: Universidad de León (2003). 1a–130a.

[41] Aranceta J Serra-Majem L Arija-Val V Gil-Hernández A Martínez de Vitoria E Ortega-Anta R. Objetivos nutricionales para la población española. Consenso de la Sociedad Española de Nutrición Comunitaria 2011. Revista Española de Nutrición Comunitaria. (2011) 17:178–9.

[42] Cuervo M Baladia E Goñi L Corbalán M Manera M Basulto J. Ingestas Dietéticas de Referencia (IDR) para la población española. FESNAD 2010 In: Fesnad JD, editor. Ingestas Dietéticas de Referencia (IDR) para la población española (Consenso FESNAD 2010). Deutsch: EUNSA (2010)

[43] Kollewe K Mauss U Krampfl K Petri S Dengler R Mohammadi B. ALSFRS-R score and its ratio: a useful predictor for ALS-progression. J Neurol Sci. (2008) 275:69–73. doi: 10.1016/j.jns.2008.07.016 18721928

[44] Salas-Salvadó J Rubio MA Barbany M Moreno B Colaborativo G de la Seedo. Consensus for the evaluation of overweight and obesity and the establishment of therapeutic intervention criteria. Med Clin. (2007) 128:184–6. doi: 10.1016/s0025-7753(07)72531-9 17298782

[45] Burgos R Bretón I Cereda E Desport JC Dziewas R Genton L. ESPEN guideline clinical nutrition in neurology. Clin Nutr. (2018) 37:354–6. doi: 10.1016/j.clnu.2017.09.003 29274834

[46] Gantenbien KV Kanata-Gantenbien C. Mediterranean diet as an antioxidant: the impact on metabolic health and overall wellbeing. Nutrients. (2021) 13:1951. doi: 10.3390/nu13061951 34204057 PMC8227318

[47] Kellogg J Bottman L Arra EJ Selkirk SM Kozlowski F. Nutrition management methods effective in increasing weight, survival time and functional status in ALS patients: a systematic review. Amyotr Late Scler Frontotemp Degener. (2018) 19:7–11. doi: 10.1080/21678421.2017.1360355 28799809

[48] Paganoni S Deng J Jaffa M Cudkowicz ME Wills AM. Body mass index, not dyslipidemia, is an independent predictor of survival in amyotrophic lateral sclerosis. Muscle Nerve. (2011) 44:20–4. doi: 10.1002/mus.22114 21607987 PMC4441750

[49] Héritier AC Janssens JP Adler D Ferfoglia RI Genton L. Should patients with ALS gain weight during their follow-up? Nutrition. (2015) 31:1368–71. doi: 10.1016/j.nut.2015.06.005 26429657

[50] Romero-Corral A Somers VK Sierra-Johnson J Thomas RJ Collazo-Clavell ML Korinek J. Accuracy of body mass index in diagnosing obesity in the adult general population. Int J Obes. (2008) 32:959–6. doi: 10.1038/ijo.2008.11 PMC2877506 18283284

[51] You T Nicklas BJ. Chronic inflammation: role of adipose tissue and modulation by weight loss. Curr Diabetes Rev. (2006) 2:29–37. doi: 10.2174/157339906775473626 18220615

[52] Boghossian NS Yeung EH Mumford SL Zhang C Gaskins AJ Wactawski-Wende J. Adherence to the Mediterranean diet and body fat distribution in reproductive aged women. Eur J Clin Nutr. (2013) 67:289–4. doi: 10.1038/ejcn.2013.4 23388669 PMC3594052

[53] Meydani M Hasan ST. Dietary polyphenols and obesity. Nutrients. (2010) 2:737–1. doi: 10.3390/nu2070737 22254051 PMC3257683

[54] Gómez-Zorita S Belles C Briot A Fernández-Quintela A Portillo MP Carpéné C. Pterostilbene inhibits Lipogenic activity similar to resveratrol or caffeine but differently modulates lipolysis in adipocytes. Phytother Res. (2017) 31:1273–82. doi: 10.1002/ptr.5852 28627722

[55] Liu Y Zhang Y Xu Q Yu X Zhang X Wang J. Increased norepinephrine by medium-chain triglyceride attributable to lipolysis in white and brown adipose tissue of C57BL/6J mice. Biosci Biotechnol Biochem. (2012) 76:1213–8. doi: 10.1271/bbb.120079 22790949

[56] Dinicolantonio JJ O’Keefe JH. Good fats versus bad fats: a comparison of fatty acids in the promotion of insulin resistance, inflammation, and obesity. Mo Med. (2017) 114:303–7. 30228616 PMC6140086

[57] Lee I Kazamel M McPherson T McAdam J Bamman M Amara A. Fat mass loss correlates with faster disease progression in amyotrophic lateral sclerosis patients: exploring the utility of dual-energy x-ray absorptiometry in a prospective study. PLoS One. (2021) 16:e0251087. doi: 10.1371/journal.pone.0251087 33956876 PMC8101939

[58] Scaricamazza S Salvatori I Ferri A Valle C. Skeletal muscle in ALS: an unappreciated therapeutic opportunity? Cells. (2021) 10:525. doi: 10.3390/cells10030525 33801336 PMC8000428

[59] Frederick DW Loro E Liu L Davila A Chellappa K Silverman IM. Loss of NAD homeostasis leads to progressive and reversible degeneration of skeletal muscle. Cell Metab. (2016) 24:269–2. doi: 10.1016/j.cmet.2016.07.005 27508874 PMC4985182

[60] Conley KE Jubrias SA Esselman PC. Oxidative capacity and ageing in human muscle. J Physiol. (2000) 526:203–13. doi: 10.1111/j.1469-7793.2000.t01-1-00203.x 10878112 PMC2269983

[61] De Guia RM Agerholm M Nielsen TS Consitt LA Søgaard D Helge JW. Aerobic and resistance exercise training reverses age-dependent decline in NAD^+^ salvage capacity in human skeletal muscle. Physiol Rep. (2019) 7:e14139. doi: 10.14814/phy2.14139 31207144 PMC6577427

[62] Granic A Sayer AA Robinson SM. Dietary patterns, skeletal muscle health, and sarcopenia in older adults. Nutrients. (2019) 11:745. doi: 10.3390/nu11040745 30935012 PMC6521630

[63] Tian HY Qiu R Jing LP Chen ZY Chen GD Chen YM. Alternate Mediterranean diet score is positively associated with skeletal muscle mass index in middle-aged adults. Br J Nutr. (2017) 117:1181–8. doi: 10.1017/S0007114517001118 28514984

[64] Korrapati D Jeyakumar SM Putcha UK Mendu VR Ponday LR Acharya V. Coconut oil consumption improves fat-free mass, plasma HDL-cholesterol and insulin sensitivity in healthy men with normal BMI compared to peanut oil. Clin Nutr. (2019) 38:2889–99. doi: 10.1016/j.clnu.2018.12.026 30630708

[65] Johnstone AM Murison SD Duncan JS Rance KA Speakman JR. Factors influencing variation in basal metabolic rate include fat-free mass, fat mass, age, and circulating thyroxine but not sex, circulating leptin, or triiodothyronine. Am J Clin Nutr. (2005) 82:941–8. doi: 10.1093/ajcn/82.5.941 16280423

[66] Volek JS Phinney SD Forsythe CE Quann EE Wood RJ Puglisi MJ. Carbohydrate restriction has a more favorable impact on the metabolic syndrome than a low fat diet. Lipids. (2009) 44:297–9. doi: 10.1007/s11745-008-3274-2 19082851

[67] Weerasekera A Sima DM Dresselaers T Van Huffel S Van Damme P Himmelreich U. Non-invasive assessment of disease progression and neuroprotective effects of dietary coconut oil supplementation in the ALS SOD1G93A mouse model: a 1H-magnetic resonance spectroscopic study. Neuro Image Clinical. (2018) 20:1092–05. doi: 10.1016/j.nicl.2018.09.011 PMC6202692 30368196

[68] Benlloch M López-Rodríguez MM Cuerda-Ballester M Drehmer E Carrera S Ceron JJ. Satiating effect of a ketogenic diet and its impact on muscle improvement and oxidation state in multiple sclerosis patients. Nutrients. (2019) 11:E1156. doi: 10.3390/nu11051156 PMC6566517 31126118

[69] Platero JL Cuerda-Ballester M Sancho-Cantus D Benlloch M Ceron JJ Peres Rubio C. The impact of epigallocatechin Gallate and coconut oil treatment on cortisol activity and depression in multiple sclerosis patients. Life. (2021) 11:353. doi: 10.3390/life11040353 33920655 PMC8073508

[70] Jennings A Mulligan AA Khaw KT Luben RN Welch AA. A Mediterranean diet is positively associated with bone and muscle health in a non-Mediterranean region in 25,450 men and Women from EPIC-Norfolk. Nutrients. (2020) 12:1154. doi: 10.3390/nu12041154 32326165 PMC7231007

[71] Roubeau V Blasco MF Corcia P Praline J. Nutritional assessment of amyotrophic lateral sclerosis in routine practice: value of weighing and bioelectrical impedance analysis. Muscle Nerve. (2015) 51:479–4. doi: 10.1002/mus.24419 25130859

[72] Jin-Yue L Xiao-Han S Zheng-Yi C Dong-Chao S Xun-Zhe Y Ming-Sheng L. Correlation of weight and body composition with disease progression rate in patients with amyotrophic lateral sclerosis. Sci Rep. (2022) 12:13292. doi: 10.1038/s41598-022-16229-9 35918363 PMC9345931

[73] Ah B Pe S. Adipose tissue, inflammation, and cardiovascular disease. Circ Res. (2005) 96:939–49. doi: 10.1161/01.RES.0000163635.62927.34 15890981

[74] Perry VH Cunningham C Holmes C. Systemic infections and inflammation affect chronic neurodegeneration. Nat Rev Immunol. (2007) 7:161–7. doi: 10.1038/nri2015 17220915

[75] Perry VH Nicoll JAR Holmes C. Microglia in neurodegenerative disease. Nat Rev Neurol. (2010) 6:193–1. doi: 10.1038/nrneurol.2010.17 20234358

[76] Dahlke C Saberi D Ott B Brand-Saberi B Schmitt-John T Theiss C. Inflammation and neuronal death in the motor cortex of the wobbler mouse, an ALS animal model. J Neuroinflammation. (2015) 12:215. doi: 10.1186/s12974-015-0435-0 26597538 PMC4657283

[77] Yudkin JS. Adipose tissue, insulin action and vascular disease: inflammatory signals. Int J Obes Relat Metab Disord. (2003) 27:25–8. doi: 10.1038/sj.ijo.0802496 14704740

[78] Nevill AM Stewart AD Olds T Duncan MJ. A new waist-to-height ratio predicts abdominal adiposity in adults. Res Sports Med. (2020) 28:15–26. doi: 10.1080/15438627.2018.1502183 30044132

[79] Leclerc AF Foidart-Dessalle M Tomasella M Coucke P Devos M Bruyère O. Multidisciplinary rehabilitation program after breast cancer: benefits on physical function, anthropometry and quality of life. Eur J Phys Rehabil Med. (2017) 53:633–2. doi: 10.23736/S1973-9087.17.04551-8 28322035

[80] Román E García-Galcerán C Torrades T Herrera S Marín A Doñate M. Effects of an exercise Programme on functional capacity, body composition and risk of Falls in patients with cirrhosis: a randomized clinical trial. PLoS One. (2016) 11:e0151652. doi: 10.1371/journal.pone.0151652 27011355 PMC4807034

[81] de Oliveira SA Dutra MT de Moraes WMAM Funghetto SS Lopes de Farias D Dos Santos PHF. Resistance training-induced gains in muscle strength, body composition, and functional capacity are attenuated in elderly women with sarcopenic obesity. Clin Interv Aging. (2018) 13:411–7. doi: 10.2147/CIA.S156174 29588579 PMC5858549

[82] Ioannides ZA Steyn FJ Henderson RD Mccombe PA Ngo ST. Anthropometric measures are not accurate predictors of fat mass in ALS. Amyotr Late Scler Frontotemp Degener. (2017) 18:486–1. doi: 10.1080/21678421.2017.1317811 28446030

